# Bottom‐up Fabrication and Atomic‐Scale Characterization of Triply Linked, Laterally π‐Extended Porphyrin Nanotapes**

**DOI:** 10.1002/anie.202105350

**Published:** 2021-06-14

**Authors:** Qiang Sun, Luis M. Mateo, Roberto Robles, Nicolas Lorente, Pascal Ruffieux, Giovanni Bottari, Tomás Torres, Roman Fasel

**Affiliations:** ^1^ Departamento de Química Orgánica Universidad Autónoma de Madrid Campus de Cantoblanco 28049 Madrid Spain; ^2^ IMDEA-Nanociencia Campus de Cantoblanco 28049 Madrid Spain; ^3^ Institute for Advanced Research in Chemical Sciences (IAdChem) Universidad Autónoma de Madrid 28049 Madrid Spain; ^4^ nanotech@surfaces Laboratory Empa-Swiss Federal Laboratories for Materials Science and Technology 8600 Dübendorf Switzerland; ^5^ Centro de Física de Materiales CFM/MPC (CSIC-UPV/EHU) Paseo de Manuel de Lardizabal 5 20018 Donostia-San Sebastián Spain; ^6^ Donostia International Physics Center (DIPC) 20018 Donostia-San Sebastián Spain; ^7^ Materials Genome Institute Shanghai University 200444 Shanghai China; ^8^ Department of Chemistry and Biochemistry University of Bern 3012 Bern Switzerland

**Keywords:** on-surface synthesis, open-shell, porphyrin nanotapes, scanning probe microscopy/spectroscopy, spin-split end states

## Abstract

Porphyrin nanotapes (Por NTs) are promising structures for their use as molecular wires thanks to a high degree of π‐conjugation, low HOMO—LUMO gaps, and exceptional conductance. Such structures have been prepared in solution, but their on‐surface synthesis remains unreported. Here, meso–meso triply fused Por NTs have been prepared through a two‐step synthesis on Au(111). The diradical character of the on‐surface formed building block **PorA_2_**, a phenalenyl π‐extended Zn^II^Por, facilitates intermolecular homocoupling and allows for the formation of laterally π‐extended tapes. The structural and electronic properties of individual Por NTs are addressed, both on Au(111) and on a thin insulating NaCl layer, by high‐resolution scanning probe microscopy/spectroscopy complemented by DFT calculations. These Por NTs carry one unpaired electron at each end, which leads to magnetic end states. Our study provides a versatile route towards Por NTs and the atomic‐scale characterization of such tapes.

## Graphical abstract

Herein, we describe the first on‐surface preparation of porphyrin nanotapes (Por NTs) on a gold surface. Structural and electronic characterization reveals that the Por NTs carry one unpaired electron at each end, which leads to magnetic end states. This study provides an alternative and versatile route to the fabrication of Por NTs and a detailed atomic‐scale characterization of their structural and electronic properties.

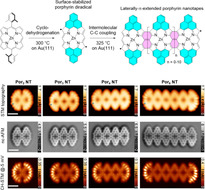

## Introduction

Tetrapyrroles, “the pigments of life”, are key molecules for the metabolism of living organisms, supporting functions of vital importance such as electron transport, light‐harvesting and oxygen reduction. Within this family of compounds, porphyrins (Pors) are of particular interest thanks to their planar structure with an aromatic core of 18 π‐electrons, remarkable thermal stability, tunable redox properties, and intense optical features.^[1]^ Taking advantage of these properties and the Pors’ extraordinary chemical versatility, these macrocycles have been tailored for their use in a wide range of fields, such as photovoltaics,^[2]^ catalysis^[3]^ and molecular electronics,^[4]^ to mention a few.

In the field of organic semiconductors and molecular electronics, π‐extended Por monomers and, in particular, oligomers, have gained considerable attention as potential molecular wires,^[5]^ near‐infrared absorbers, and nonlinear optical components due to their low highest occupied molecular orbital (HOMO)–lowest unoccupied molecular orbital (LUMO) gaps arising from their large π‐conjugated structure.^[6]^ Furthermore, the inner cavity of Pors can chelate transition metal ions which allows for the construction of magnetically active nanostructures, such as organic spin filters.^[7]^

Focusing on Por oligomers, it has been demonstrated that the nature of the Por‐Por linkage dramatically affects the electronic properties.^[8]^ In particular, the number (that is, singly, doubly, or triply connected)^[9]^ and the site (i.e., β‐β, *meso*‐β, and/or *meso*‐*meso*) of the inter‐Por connectivity^[10]^ as well as the chemical nature of the linker (e.g., C−C single bond, and/or π‐spacers)^[11]^ have been identified as key factors. In this context, it was shown that the highest degree of π‐conjugation and lowest HOMO–LUMO gaps were achieved by the *meso*‐*meso*, β‐β, β‐β triple linkage, which leads to planar, fused Por oligomers.^[12]^ Such triply fused Por oligomers show remarkable transport properties[^5b^, ^13^] compared to typical “benchmark” systems like oligophenylenevinylene,^[14]^ oligothiophene^[15]^ and oligoyne.^[16]^

**Figure 1:**
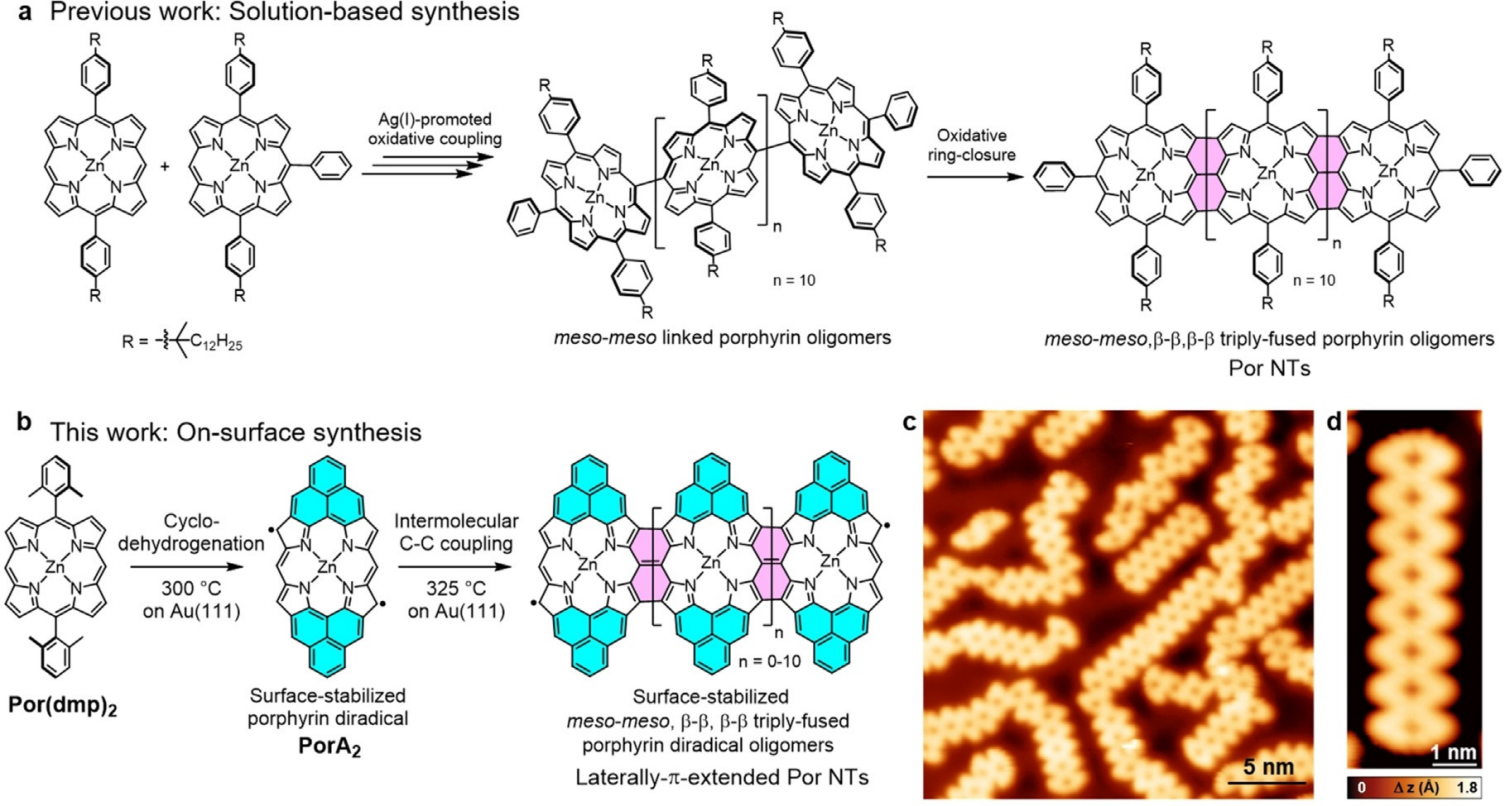
a) Solution‐based multi‐step synthesis of Por NTs as reported by Osuka and co‐workers.^[12a]^ b) Two‐step, on‐surface synthesis of laterally π‐extended Por NTs on Au(111). c) Overview STM image of the on‐surface synthesis of Por NTs (*V*_s_=−0.5 V, *I*_t_=40 pA), d) Zoom‐in STM image of **Por_8_ NT** (*V*_s_=−0.06 V, *I*_t_=160 pA).

Up to date, triply fused Pors have been obtained in solution through oxidative ring closure of their corresponding *meso*‐*meso* linked precursor oligomers bearing long alkyl chains and/or bulky substituents^[12b]^ at some Por *meso*‐positions. Using this strategy, Osuka et al. reported triply fused Por dimers and trimers, dodecamers (Figure 1 a),^[12a]^ and longer oligomers containing up to 24 Por units,^[12b]^ all of them obtained after a long multi‐step synthesis in extremely low overall yields. Moreover, the low HOMO–LUMO gap of the resulting Por tapes led to severe stability issues, especially for the longest oligomers. Additionally, the π‐stacking tendency of these conjugates dramatically increases upon increasing the number of Por units, leading to solubility problems.

In the last decade, on‐surface synthesis on atomically clean surfaces under ultra‐high vacuum (UHV) conditions has emerged as an appealing alternative for the fabrication of planar, poorly soluble and inherently unstable π‐conjugated systems.^[17]^ Moreover, the resulting on‐surface synthesized nanostructures can be directly accessed by local‐probe techniques such as scanning probe microscopy allowing for a detailed “in situ” structural and electronic characterization with molecular or even chemical bond resolution.^[18]^

In the context of on‐surface Por chemistry, many different systems have been prepared and studied, mainly involving one‐dimensional (1D) and two‐dimensional (2D) networks and their topological aspects.^[19]^ Regarding triply fused Por systems, only porphine dimers^[20]^ and Por dimers spaced by short graphene nanoribbon (GNR) segments^[21]^ have been reported. Meanwhile, the surface‐assisted synthesis of triply fused Por oligomers remains challenging and has not been achieved to date.

Herein, a simple, two‐step strategy for the on‐surface synthesis of laterally π‐extended Por nanotapes (NTs) is presented (Figure 1 a). The structural and electronic properties of individual Por NTs, both on Au(111) and NaCl/Au(111), have been scrutinized by high‐resolution scanning probe microscopy/spectroscopy complemented with density functional theory (DFT) calculations. Remarkably, the fabricated Por NTs feature magnetic end states resulting from the presence of an unpaired electron at each end of the Au(111)‐supported NTs.

## Results and Discussion

The synthetic route employed for the fabrication of surface‐supported laterally π‐extended Por NTs starts with **Por(dmp)_2_** which was synthesized in few steps by solution chemistry.^[22]^ Sublimation of the latter Por on clean Au(111) under UHV conditions followed by thermal activation at 300 °C afforded two‐fold phenalenyl‐fused Por derivative **PorA_2_** via surface‐assisted cyclodehydrogenation.^[22]^

**PorA_2_** exhibits a diradical open‐shell character with two unpaired electrons delocalized over the Por longer “edges”. Despite their delocalization, the reactivity of these radicals is particularly high at the Por β‐pyrrolic positions, as demonstrated by the formation of β‐hydrogenated Por species.^[22]^ Taking into account that surface‐stabilized radicals are key intermediates for the on‐surface Ullmann‐type coupling reaction, we decided to explore the possible thermally activated polymerization of **PorA_2_**. Indeed, further annealing of **PorA_2_** at 325 °C triggered the formation of oligomeric Por species, namely Por NTs, in which **PorA_2_** units are *meso*‐*meso*, β‐β, β‐β triply fused (Figure 1 b).

A typical STM image of a sample prepared through this two‐step annealing is shown in Figure 1 c, and reveals several linear, *meso*‐*meso*, β‐β, β‐β triply fused Por NTs such as a **Por_8_ NT** (Figure 1 d). Longer tapes containing up to 12 triply fused Pors (**Por_12_ NT**) could also be obtained (Supporting Information, Figure S1.0). Moreover, the presence of some “ill‐formed” Por NTs is observed, resulting from the fusion between the phenalenyl edge and the Por edge and/or the β‐*meso* Por linkage, both of which arise from the delocalized nature of the radical. In the following, we focus exclusively on the main products, the linearly fused, regular Por NTs.

**Figure 2:**
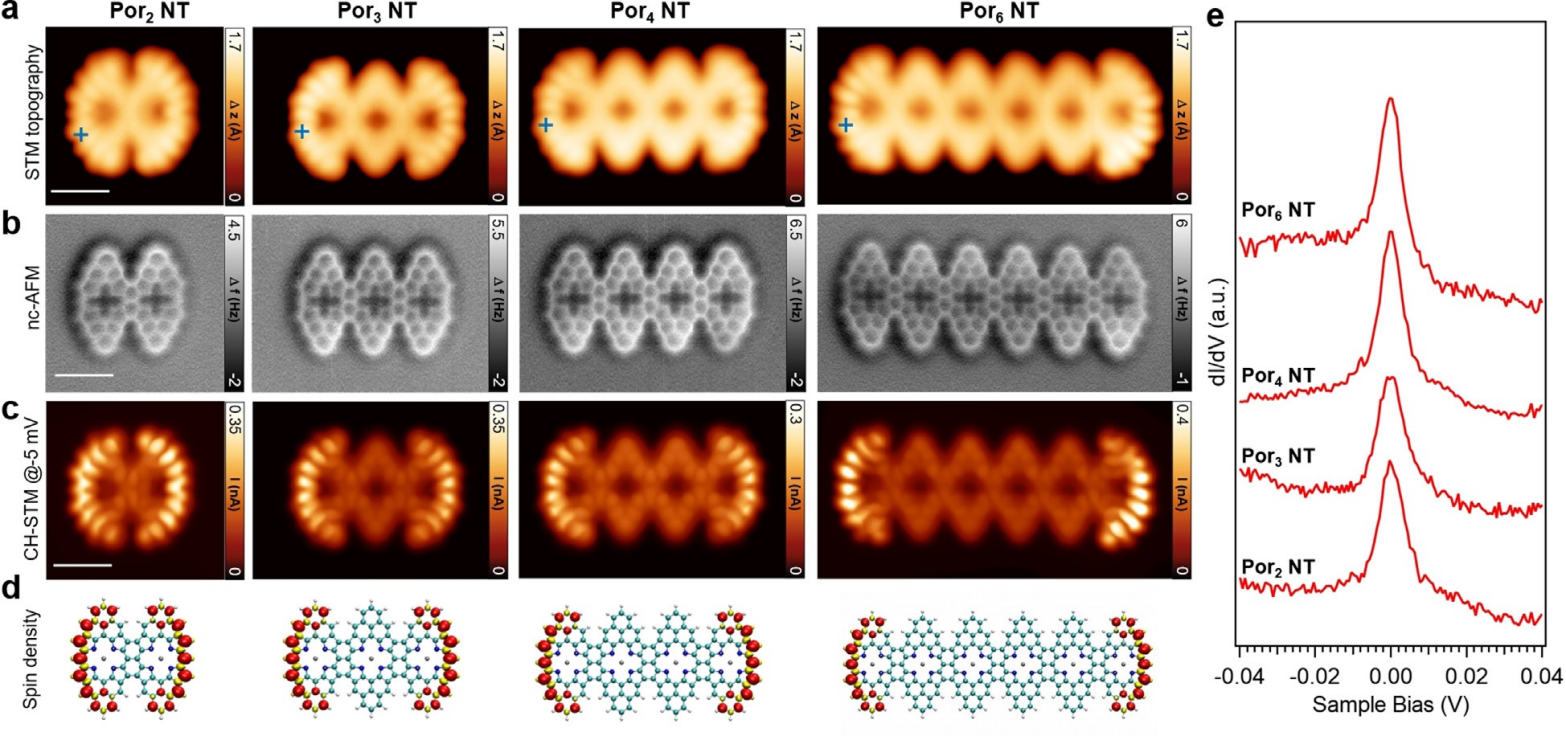
a) Topographic STM images, b) constant‐height nc‐AFM, and c) simultaneously acquired constant‐height STM image (*V*_s_=−0.005 V) showing the spatial distribution of the Kondo resonance for **Por_2_ NT**, **Por_3_ NT**, **Por_4_ NT**, and **Por_6_ NT**, respectively. A CO‐functionalized tip was used. Set points: a) from left to right: *V*_s_=−0.06 V, *I*_t_=100 pA; *V*_s_=−0.06 V, *I*_t_=220 pA; *V*_s_=−0.06 V, *I*_t_=220 pA; *V*_s_=−0.1 V, *I*_t_=120 pA. Scale bars: 1 nm. d) DFT computed spin densities of Por NTs imaged in (a), e) d*I*/d*V* spectra acquired at the end of the Por NTs imaged in (a) (blue crosses), revealing Kondo resonances for all the tapes (Set points: *V*_s_=−0.06 V, *I*_t_=300 pA, *V*_mod_=1 mV).

High‐resolution STM and bond‐resolved nc‐AFM imaging^[18]^ have been carried out for a series of Por NTs with different numbers of constituting Por units (Figures 2 a and b). The corresponding nc‐AFM images unequivocally prove the triple fusion between adjacent Pors in the NTs by evidencing the formation of three new C−C bonds between neighboring Por units. As demonstrated in our earlier work, the phenalenyl moieties confer an open‐shell character to **PorA_2_**, which gives rise to low‐bias spectroscopy features.^[22]^

Therefore, the question arises whether the open‐shell character is preserved for oligomeric Por NTs. To address this question, chemical structure analysis was initially performed, which suggests that, as in the case of **PorA_2_**, no Kekulé resonance structures can be drawn for Por NTs (Supporting Information, Figure S1.4). Among the possible resonance structures, the one which shows the maximum number of Clar sextets and minimum number of unpaired electrons (i.e., 2), as the one drawn in Figure 1 b and the Supporting Information, Figure S1.4, can be expected to contribute most to the electronic structure.

In analogy to **PorA_2_**, the unpaired electrons in Por NTs give rise to a higher reactivity, making the termini susceptible to hydrogenation,^[22]^ which can be demonstrated by nc‐AFM imaging thanks to the extreme sensitivity of this technique to the apparent height of the adsorbates.^[23]^ Indeed, a close inspection of the Por tapes by constant‐height nc‐AFM imaging reveals doubly hydrogenated carbon atoms (CH_2_) at the terminal β positions of some of the tapes (Supporting Information, Figure S1.1). Using the same STM tip induced atomic manipulation protocol as for the hydrogenated **PorA_2_** monomer,^[22]^ we could selectively transform the terminal C(sp_3_)H_2_ into C(sp_2_)H, thereby removing the “extra” hydrogen atom (Figure S1.1).

**Figure 3:**
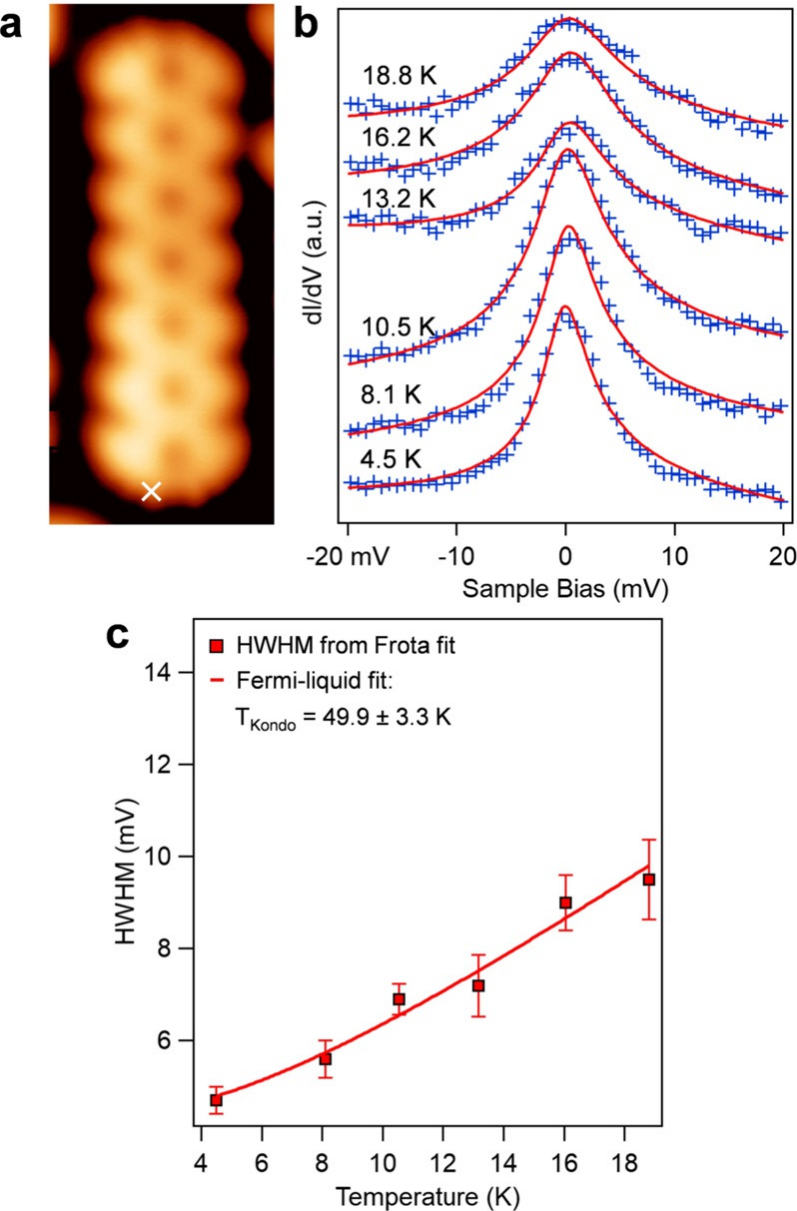
a) STM image of **Por_7_ NT** (*V*_s_=−0.05 V, *I*_t_=320 pA), b) Temperature evolution of the Kondo spectra, with the experimental data (blue crosses) fit by the Frota function (red curves). Data have been acquired at the position marked by a cross in (a) (*V*_mod_=0.8 mV). c) Extracted half width at half maximum (HWHM) of the Kondo resonance as a function of temperature. The data are fit by the Fermi‐liquid model to determine the Kondo temperature.

The unpaired electron at each end of the finite Por NTs indicates a localized spin S=1/2 , which may give rise to a many‐body Kondo resonance originating from the screening of the localized spin by itinerant electrons from the underlying metal surface.^[24]^ To explore this scenario, we have performed differential conductance (d*I*/d*V*) spectroscopy over Por NTs of different lengths (Figures 2 e and 3). Sharp resonances around zero bias are indeed observed for all the tapes investigated pointing at the presence of the Kondo effect. We find that this zero‐bias resonance is suppressed upon hydrogenation of the corresponding terminal β position, further confirming the S=1/2 nature of the spin at the edge (Supporting Information, Figure S1.1c).

Constant‐height STM imaging at −5 mV reveals the spatial distribution of the Kondo resonance which is localized over both ends of the Por tapes (Figure 2 c). To gain further insight into the magnetic properties of the Por NTs, we have performed spin‐polarized DFT calculations of free‐standing Por NTs, which also demonstrate a spin‐polarized ground state of the tapes with a net magnetic moment of 1 μ_B_ (S=1/2 ) at each end. The DFT‐computed spin density maps of the corresponding tapes are displayed in Figure 2 d, and show excellent agreement with the experimental Kondo maps in Figure 2 c. The computed exchange coupling strength decreases with increasing length of the tapes, from 1.3 meV for **Por_2_ NT**, 0.2 meV for **Por_3_ NT**, to below the computational precision for longer Por NTs. We do not observe any inelastic spin excitation next to the Kondo resonance as previously reported in similar systems with exchange‐coupled spins,^[22]^ which we attribute to the relatively broad Kondo peak and the low exchange coupling strength of 1.3 meV or less as evaluated from broken‐symmetry solutions of the DFT calculations.

The observed Kondo resonances can be nicely fit with a Frota function,^[25]^ from which the resonance width (half width at half maximum, HWHM) can be determined (Figure 3 b). The energy scale of the Kondo effect is typically expressed by the Kondo temperature *T*_K_, which is directly related to the width of the Kondo resonance. At lower temperatures (*T*<*T*_k_), the Kondo resonance lies in the strong coupling regime and exhibits characteristic temperature‐dependent broadening following the Fermi‐liquid model.^[26]^

In Figure 3 b,c, we report the temperature evolution of the Kondo resonance at the end of **Por_7_ NT**. By fitting the HWHM *Γ* of the resonances with the Fermi‐liquid model $\Gamma =\frac{1}{2}\sqrt{{\left(\alpha k_{B}T\right)}^{2}+{\left(2k_{B}T_{K}\right)}^{2}}$ , we obtained a Kondo temperature *T*_K_=49.9±3.3 K, and a multiplicative factor *α*=10.8±0.4.

1D organic structures with unpaired electrons and thus localized spins at their termini have also been observed in armchair graphene nanoribbons (AGNRs) with zigzag type ends.^[27]^ When these AGNRs are directly adsorbed on the metal surface, charge transfer to the metal empties the singly occupied end states. However, the termini of AGNRs with a transverse width of 7 carbon atoms (7AGNR) clearly display spin‐split end states after being transferred onto a thin insulating film (“decoupling layer”).^[27]^

5AGNRs are also predicted to host localized magnetic moments at their termini, and a zero‐bias resonance in STM‐based transport spectra has indeed been detected for 5AGNRs with one of their termini located on a decoupling layer.^[28]^ Furthermore, localized end states featuring zero‐bias resonances have been observed for an ethynylene‐bridged anthracene polymer.^[29]^

Such localized end states are gaining increasing interest due to their origin in topologically non‐trivial electronic quantum phases, and because they may find applications in future quantum devices.^[30]^

Owing to electron correlations, the unpaired electrons at the two ends of the Por NTs are expected to result in spin‐polarized singly occupied and unoccupied molecular orbitals (SOMO and SUMO, respectively).^[31]^ To prevent orbital hybridization with the underlying metal surface (and thus to enable probing of intrinsic electronic properties), a common strategy is to intercalate a thin insulating film between the molecules and the metal surface.[^27^, ^28^, ^31^]

**Figure 4:**
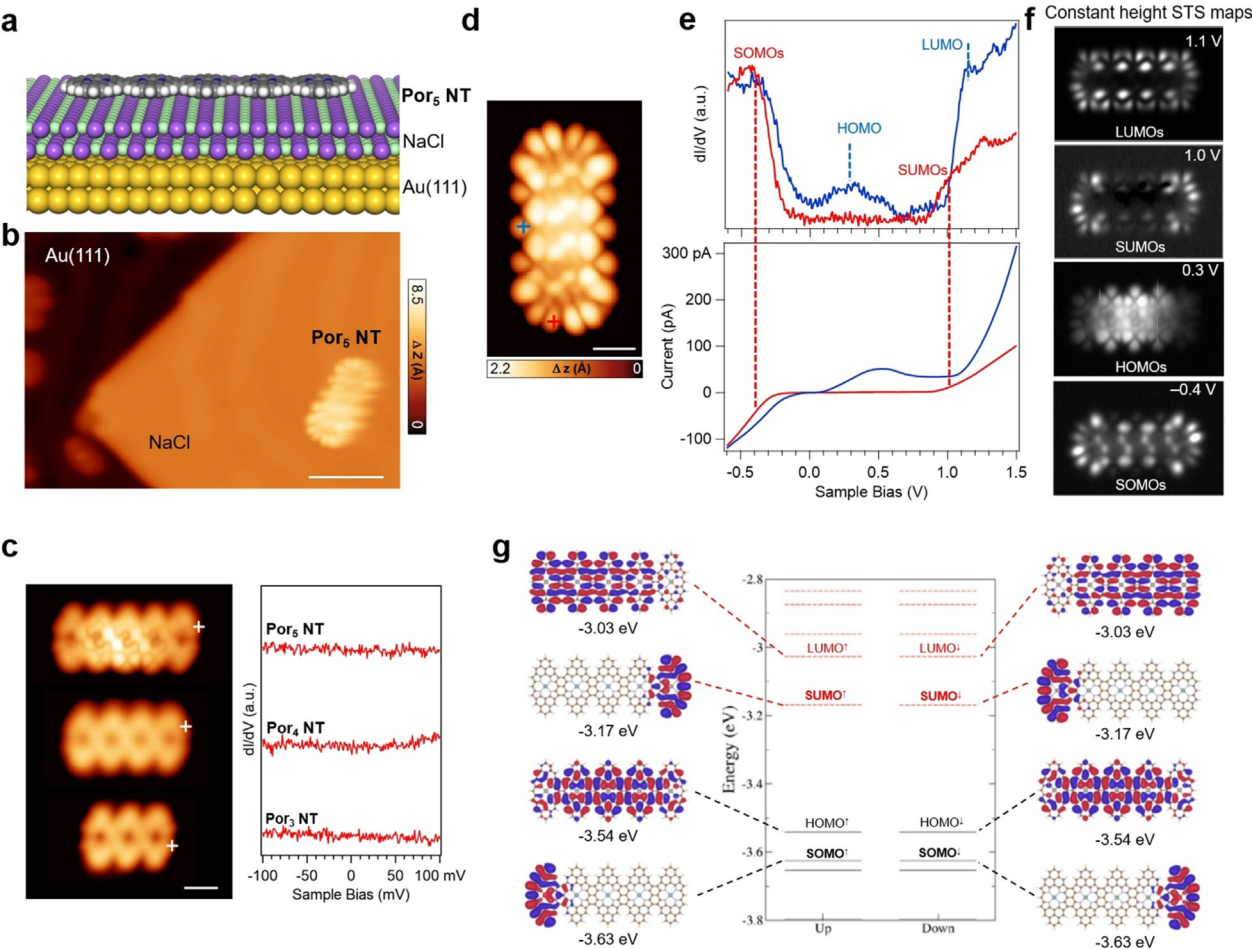
a) Model illustrating **Por_5_ NT** on a thin insulating NaCl bilayer supported by Au(111). b) STM image of **Por_5_ NT** transferred onto NaCl/Au(111) (*V*_s_=−0.2 V, *I*_t_=12 pA). Scale bar: 4 nm. c) (Left) STM images of **Por_5_ NT** pentamer, **Por_4_ NT** tetramer, and **Por_3_ NT** trimer (from top to bottom) on NaCl/Au(111), and (right) the corresponding low‐energy d*I*/d*V* spectra acquired at their termini (positions indicated by white crosses in (c). Set points: for **Por_5_ NT**: *V*_s_=−0.1 V, *I*_t_=20 pA; for **Por_4_ NT**: *V*_s_=−0.1 V, *I*_t_=8 pA; for **Por_3_ NT**: *V*_s_=0.1 V, *I*_t_=6 pA. Scale bar: 1 nm. d) STM image of **Por_5_ NT** on NaCl/Au(111) (*V*_s_=−0.6 V, *I*_t_=120 pA). Scale bar: 1 nm. e) Differential conductance d*I*/d*V* and simultaneously acquired current (*I*/*V*) spectra recorded over the **Por_5_ NT** (acquisition positions indicated by crosses in (d). f) Constant‐height STS maps at different bias voltages, as indicated. Set points from top to bottom: *V*_s_=0.1 V, *I*_t_=6 pA; *V*_s_=0.1 V, *I*_t_=6 pA; *V*_s_=0.1 V, *I*_t_=6 pA; *V*_s_=−0.1 V, *I*_t_=6 pA. g) Spin‐polarized DFT calculated molecular orbitals and energy levels of **Por_5_ NT** in gas phase (the antiferromagnetic case is shown). There are two degenerate SOMO and SUMO states, which are spatially located at the ends of the tape. SOMO is found to be shifted below the HOMO of **Por_5_ NT**.

Following the formation of the Por NTs, we therefore deposited a submonolayer of NaCl onto the samples, which led to bilayer islands of (001)‐terminated NaCl on parts of the Au(111) surface. STM manipulation was thereafter applied to transfer Por NTs onto such a NaCl island (Figures 4 a and b). An enhanced contribution of electronic frontier states to STM images immediately indicates an efficient electronic decoupling of the Por NT from the metal substrate (Figure 4 d). Meanwhile, at lower bias voltages where there are no molecular states accessible, only the molecular skeletons are visible from the STM images (Figure 4 c). We have first examined the low‐bias d*I*/d*V* spectra at the ends of Por NTs of different lengths (Figure 4 c), where no Kondo‐like features are observed as expected due to the absence of conduction electrons in the underlying NaCl island.

Wide‐range d*I*/d*V* and the concomitant current spectra have been acquired on the Por NTs to access their molecular orbitals (**Por_5_ NT** in Figure 4 e, and **Por_4_ NT** in the Supporting Information, Figure S1.2). The spectra acquired at the end of the Por NTs display a broad gap region of low conductance (red line in *I*/*V* spectrum, Figure 4 e) and broad peaks at around −0.4 V and 1.0 V (red line in d*I*/d*V* spectrum, Figure 4 e), respectively. Meanwhile, the spectra taken at the central part of the Por NT exhibit three characteristic broad peaks, the first one at negative bias in proximity to that of the end, the second one at a small positive bias centered around 0.2 V, and the third one pronounced at 1.1 V.

Spin‐polarized DFT calculations of molecular orbitals and energy levels of **Por_5_ NT** in gas phase were performed to make a direct comparison to the experimental results (the frontier orbitals and their notations are indicated in Figure 4 g). From theory, two spin‐split singly occupied and unoccupied molecular orbitals (SOMO^↑^/SOMO^↓^ and SUMO^↑^/SUMO^↓^, respectively) localized at both termini of the tape are discerned. The energy difference between SOMO and SUMO states is 0.46 eV and independent of the length of the tape (Supporting Information, Figure S1.6), as is expected for a Coulomb gap (originating from electron correlation) and in contrast to what would be expected for a hybridization gap.

The SUMO is in excellent agreement with the STS map of the tape at 1 V, while due to the close energetic proximity of SOMO and HOMO‐1 the STS map at −0.4 V comprises contributions from both (Figure 4 f). Therefore, based on the d*I*/d*V* spectra acquired at the end of the tape and the STS maps at characteristic energies, we assign the states at −0.4 V and 1.0 V to the SOMO and SUMO, respectively. Moreover, the state at 1.1 V clearly derives from the LUMO (Figures 4 e–g). Finally, the state at 0.3 V can be assigned to the HOMO which is delocalized over the central part of the tape (Figures 4 f and g), suggesting that **Por_5_ NT** is (positively) charged on NaCl/Au(111), as has also been observed for short 5AGNRs on NaCl/Au(111).^[28]^ However, because the SOMO lies lower in energy than the HOMO (Figure 4 g), the spin‐split end states SOMO^↑^/SOMO^↓^ and SUMO^↑^/SUMO^↓^ are preserved despite the depopulation of the HOMO due to charge transfer. We note a somewhat asymmetric shape of the left and right parts of the tape ends in STM images, which are also present in all the STS maps, which we attribute to some degree of hybridization and the registry of the Por NT to the underlying NaCl. Hitherto reported triply linked Por NTs have shown remarkable electronic properties and low frontier orbital gaps which makes them promising candidates for molecular electronics applications.[^12a^, ^32^] To characterize the frontier orbital gaps of our Por NTs in contact with a metal electrode, we have carried out STS experiments on Por NTs adsorbed on Au(111) complemented by DFT calculations. Interestingly, our DFT calculations for a free‐standing infinite Por NT yield a gap of 0.46 eV (Supporting Information, Figure S1.3), which is higher than the one of 0.08 eV reported for a Por tape without phenalenyl π‐extension at a similar level of theory.^[32]^ Nevertheless the electronic band gap of our Por tapes is still comparably small for a 1D organic system (for comparison, the band gap of the “quasi”‐metallic 5AGNR is about 0.42 eV by DFT).^[33]^ Experimentally, the gap of **Por_6_ NT** and longer Por NTs on Au(111) is determined to be 0.9 eV (see discussion in the Supporting Information, Figure S1.5).

## Conclusion

We have reported a facile on‐surface route towards the preparation of *meso*‐*meso*, β‐β, β‐β triply linked Por NTs, which uses a surface‐stabilized π‐extended diradical **PorA_2_** as molecular building block. The atomic structure and electronic features of such surface‐supported architectures, both on Au(111) and on a thin insulating NaCl layer, were studied by means of high‐resolution scanning probe microscopy and DFT calculations. Notably, the open‐shell character of **PorA_2_** is retained in the Por NTs, which host one unpaired electron at each end.

The presented straightforward fabrication of Por NTs paves the way towards the realization of appealing surface‐supported architectures by the complexation of magnetically active metal ions within the inner Por cavity. In this context, applications such as 1D spin filters, sensors, or catalysis can be envisioned. Moreover, the Por NTs provide a scaffold that allows the arrangement of several magnetic ions at a close distance, thereby allowing their interactions. This, together with the inherent open‐shell character of the tapes, may lead to exotic physical phenomena and quantum applications, which go beyond the actively explored pure carbon‐based 1D magnetic systems.[^28^, ^29^, ^30^]

## Conflict of interest

The authors declare no conflict of interest.

## Supporting information

As a service to our authors and readers, this journal provides supporting information supplied by the authors. Such materials are peer reviewed and may be re‐organized for online delivery, but are not copy‐edited or typeset. Technical support issues arising from supporting information (other than missing files) should be addressed to the authors.

Supplementary
