# Supplementary material for: Bottom‐up Fabrication and Atomic‐Scale Characterization of Triply Linked, Laterally π‐Extended Porphyrin Nanotapes
Source: Angew Chem Int Ed Engl. 2021 Jun 14;60(29):16208–14. doi: 10.1002/anie.202105350 (PMC8361936; doi:10.1002/anie.202105350)
Supplement: Supplementary file 1 — Supplementary [file ANIE-60-16208-s001.pdf]

Angewandte Chemie
Eine Zeitschrift der Gesellschaft Deutscher Chemiker

Supporting Information

Bottom-up Fabrication and Atomic-Scale Characterization of Triply Linked, Laterally π-Extended Porphyrin Nanotapes**

Qiang Sun+, Luis M. Mateo+, Roberto Robles+, Nicolas Lorente, Pascal Ruffieux, Giovanni Bottari,* Tomás Torres,* and Roman Fasel**

anie_202105350_sm_miscellaneous_information.pdf

Materials and Methods

STM/STS and nc-AFM characterization. A commercial low-temperature STM/AFM (Scienta Omicron) system was used for sample preparation and *in situ* characterization under ultra-high vacuum conditions (base pressure below 1×10^{-10} mbar). Au(111) single crystal substrates were cleaned by standard argon sputtering and annealing cycles. Deposition of the molecular precursors was carried out by thermal evaporation from a 6-fold organic evaporator (Mantis GmbH). STM images were recorded in constant-current mode unless otherwise stated, and the d*I*/d*V* spectra and maps were recorded using the lock-in technique (U_{rms} = 20 mV if not otherwise specified). All the measurements were carried out at 4.8 K.

Theoretical method. Density functional theory calculations were performed with the VASP code[1] using the PBE exhange and correlation functional.[2] Core electrons were treated within the projector augmented method[3] and wavefunctions were expanded using a planewave basis set with an energy cutoff of 400 eV. The (111) surface of Au was simulated using a 4 layer slab and a *vacuum* gap of 18 Å. The two bottom layers of Au were kept fixed and all the other atoms were relaxed until forces were smaller than 0.02 eV/Å (0.001 eV/Å for the gas-phase calculations). A (03 × 01 × 01) k-grid mesh was used in the relaxations, which was increased to (07 × 02 × 01) for the simulations of STM images and d*I*/d*V* maps. Van der Waals interactions have been included using the Tkatchenko-Scheffler scheme.[4] STM simulated images have been simulated within the Tersoff-Hamann method[5] as described by Bocquet *et al*.[6] and implemented in the STMpw program.[7] Images of the structures and isosurfaces were plotted using VESTA.[8]

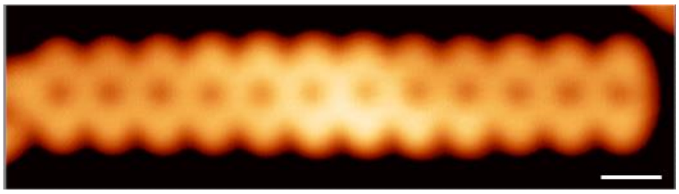

Figure S1.0 STM image of **Por_{12} NT** comprising 12 triply-fused Por units (V_s = –0.05 V, I_t = 300 pA, Scale bar: 1 nm). Note that the NT is fused to another NT at its left end.

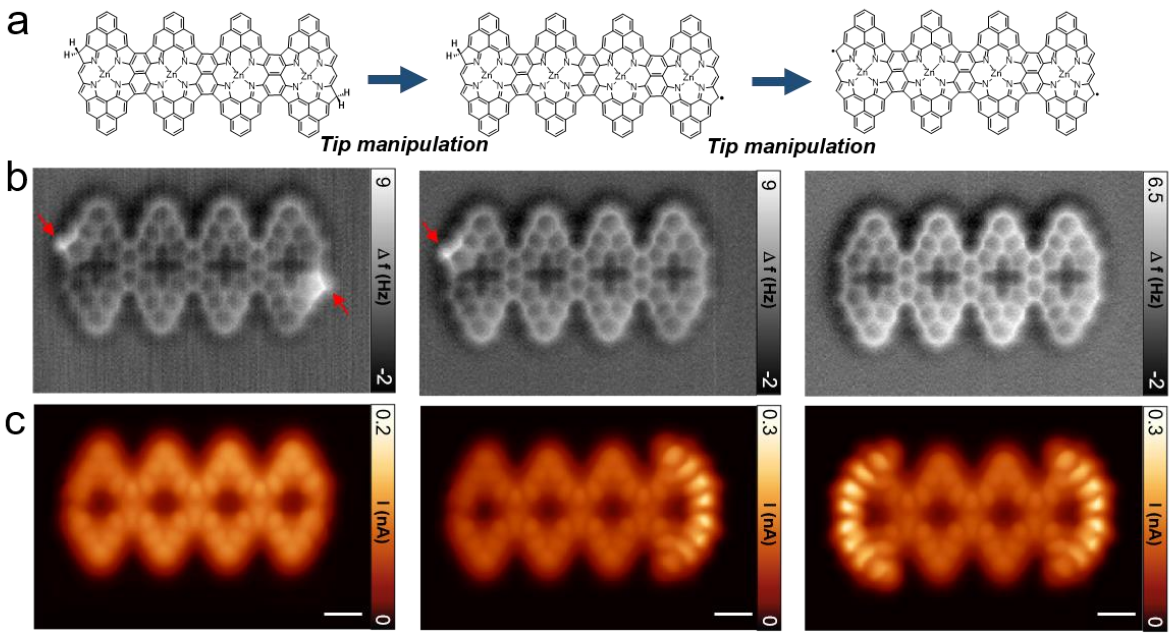

Figure S1.1 **a**, Chemical scheme of the tip-induced dehydrogenation of the CH_2 sites at the ends of **Por$_4$ NT**. **b**, Constant-height nc-AFM images of **Por$_4$ NT** before (left) and after consecutive tip-induced dehydrogenations (center and right). The arrows indicate the CH_2 structures at the *β* positions of the Por core. **c**, Constant-height STM images of the corresponding structures at – 5 mV, revealing the Kondo resonance spatially localized at the ends without CH_2 structures. Scale bars: 5 Å. The removal of the hydrogens was carried out by positioning the tip above the CH_2 site at a typical set point of V = –0.1 V and I = 10 pA, followed by a tip retraction of 4 Å. Thereafter, the bias voltage was ramped up to 3.4 eV until an abrupt change of the tunneling current occurred.

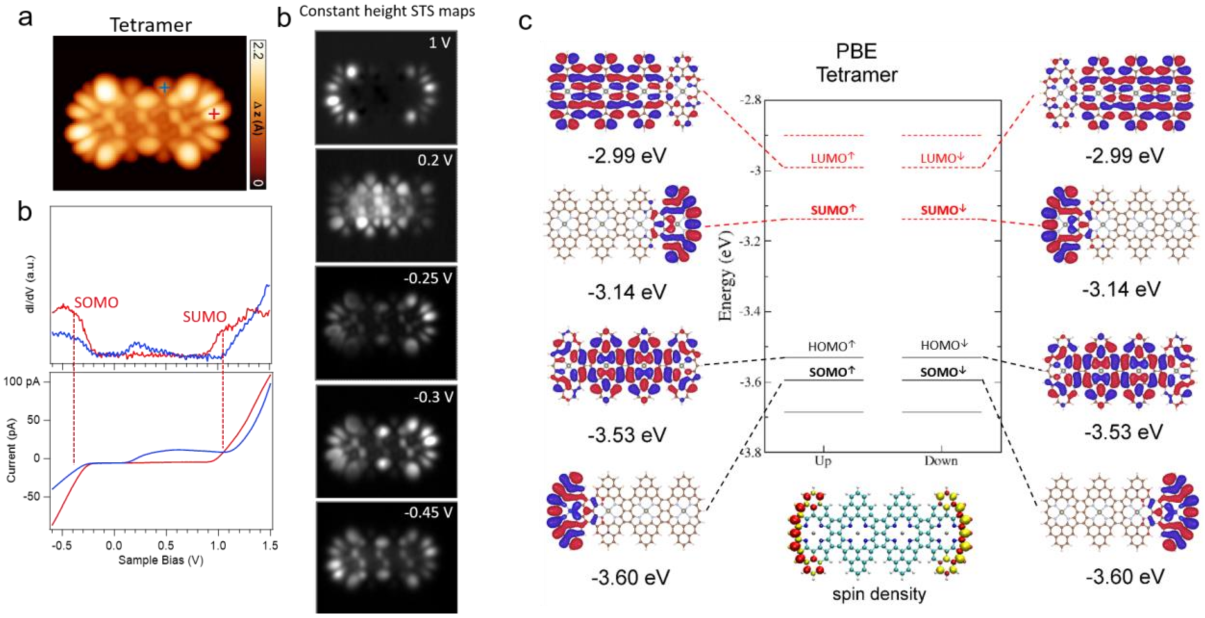

Figure S1.2 a, STM image of a decoupled **Por₄ NT** (on NaCl/Au(111), $V_s = -0.3$ V, $I_t = 30$ pA). **b**, dI/dV and simultaneously acquired current spectra recorded over the tape (spectra positions indicated by crosses in **a**). **c**, Constant-height STS maps at different bias voltages. **d**, Spin-polarized DFT calculated molecular orbitals and energy levels of **Por₄ NT** in gas phase. There are two degenerate states for both SOMO and SUMO located at the ends of the tape.

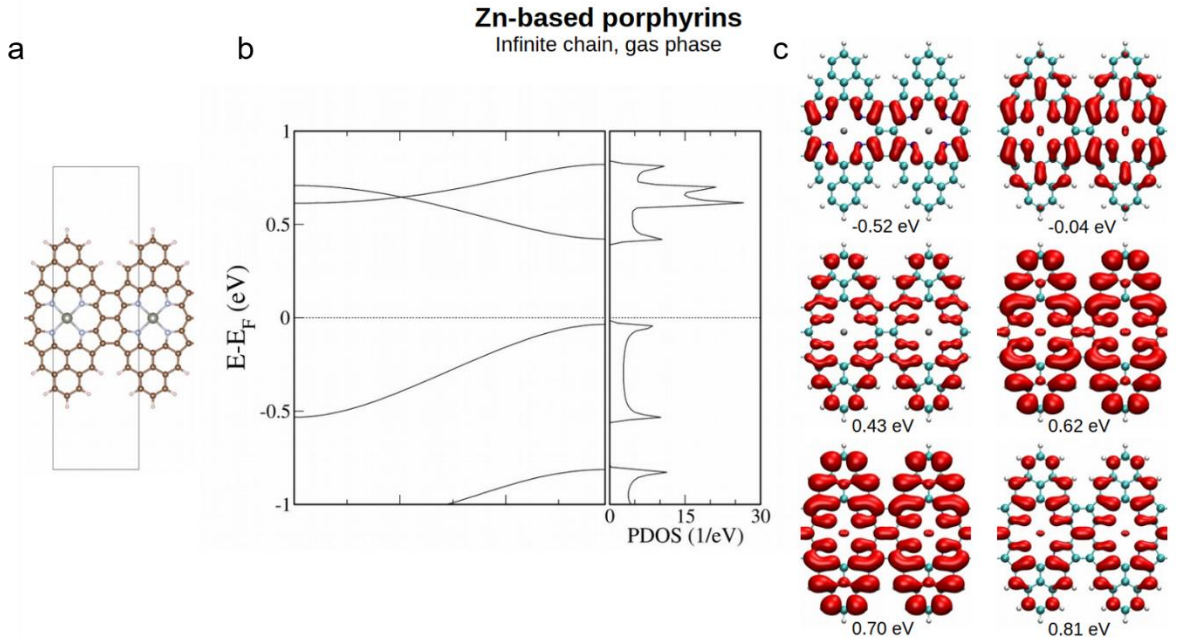

Figure S1.3 a, Model structure of an infinite Por NT in gas phase with its unit cell indicated by a rectangle. **b**, DFT calculated band structure of the infinite Por NT and its corresponding projected density of states. The band gap of the Por NT is determined to be 0.46 eV. **c**, Plots of the square of the wave functions at indicated energies.

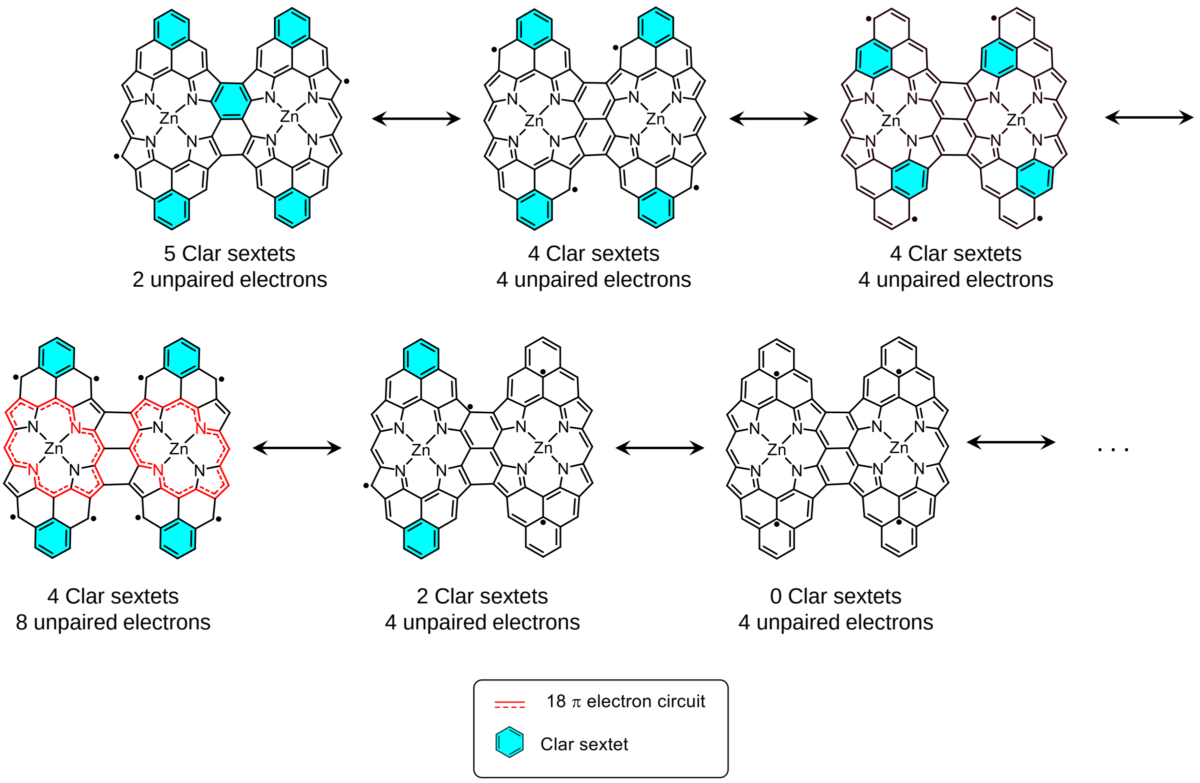

Figure S1.4 Some of the possible resonance structures of **Por$_2$ NT**.

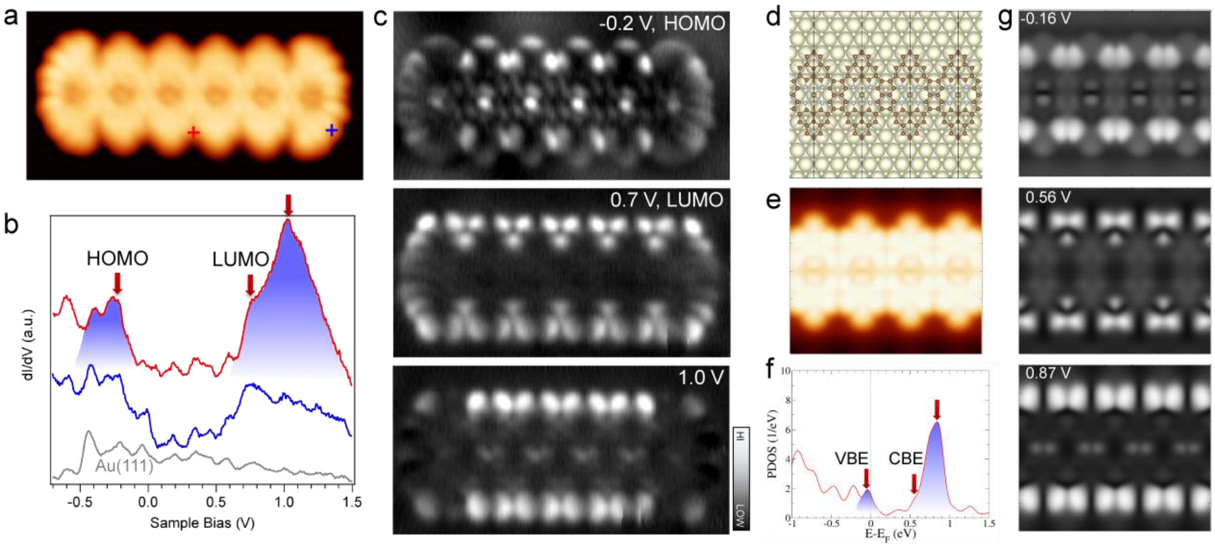

Figure S1.5 Frontier orbital gap of Por NT on Au(111). a STM image of **Por$_6$ NT** on Au(111) (V_s = –0.4 V, I_t = 220 pA). **b**, Differential conductance d*I*/d*V* spectra revealing the gap of the Por NT (V_{mod} = 20 mV) (spectrum acquisition position indicated by crosses with corresponding colors in **a**). The frontier orbitals are indicated by red arrows. **c**, Constant-current d*I*/d*V* maps of **Por$_6$ NT** at different bias voltages, with –0.2 V and 0.7 V corresponding to HOMO and LUMO, respectively. **d**, DFT optimized structural model of an infinite Por NT. The periodic unit cell is marked by black lines. **e**, DFT simulated topographic STM image of Por NT in **d**. **f**, Projected density of states (PDOS) of Por NT on Au(111) computed by DFT.

The red arrows indicate the energy positions of the valence band edge (VBE) and conduction band edge (CBE). **g**, DFT simulated d*I*/d*V* maps of the surface supported Por NT at different energies including the VBE (–0.16 V) and CBE (0.56 V), corresponding to the maps in the central parts of the finite Por NT in **c**.

Experimentally, d*I*/d*V* spectra were recorded on the central part of a finite-length Por NT, namely **Por$_6$ NT**, to access its "bulk" electronic properties on Au(111) (Figures S1.5a and S1.5b). Two broad peaks in the negative and positive bias range are discerned. By recording d*I*/d*V* maps at the characteristic bias voltages, we could clearly see the density of states distributing along the NT (Figure S1.5c). The DFT calculated projected density of states (PDOS) of an infinite Por NT on Au(111) nicely reproduces the two broad peaks at negative and positive energies, respectively (Figure S1.5f). Moreover, the simulated d*I*/d*V* maps at the corresponding energies with respect to their experimental counterparts show remarkable agreement with the "bulk" parts of the experimental d*I*/d*V* maps (Figure S1.5g). These observations suggest that the frontier orbitals of the Por NT on Au(111) are at –0.2 V and 0.7 V, respectively, and therefore a frontier orbital gap of 0.9 eV on Au(111). We note that the experimentally determined gaps of longer Por NTs are nearly the same, namely 0.9 eV for the **Por$_{12}$ NT**. We have also calculated the length evolution of the gap with DFT (Supplementary Figure S1.6). Excluding the spin-polarized SOMO and SUMO states localized at the tape ends, the bulk gap decreases from 0.71 eV for **Por$_2$ NT** to 0.50 eV for **Por$_6$ NT**, converging towards the value of the infinite Por NT (0.46 eV). If we also consider the SOMO and SUMO end states, the frontier gap decreases from 0.46 eV to 0.36 eV for **Por$_2$ NT** to **Por$_6$ NT**, respectively. We note that a qualitative comparison of these values with the experimental ones would need to take the band gap underestimation of DFT and the screening by the underlying metal substrate into account.[9]

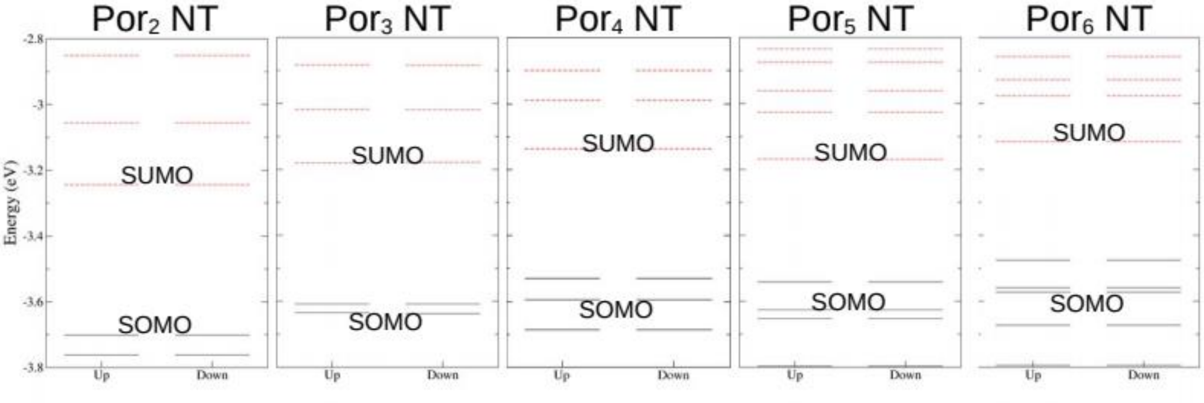

Figure S1.6 Spin-polarized DFT calculated energy levels for Por NTs in the gas phase. The spin-polarized SUMO and SOMO states are labeled. Here the spin-polarized states at the ends

are all antiferromagnetically coupled. We find that the SOMO-SUMO energy gap is constant (0.46 eV) for all tape lengths investigated, reflecting the localized nature of the spin-polarized end states. As the length of the NT increases the bulk HOMO-LUMO gap shrinks, and from **Por_3 NT** onwards the HOMO moves energetically beyond the SOMO which is no longer the most frontier orbital.

References

[1] G. Kresse, J. Furthmüller, *Comput. Mater. Sci.* **1996**, *6*, 15-50.
[2] J. P. Perdew, K. Burke, M. Ernzerhof, *Phys. Rev. Lett.* **1996**, *77,* 3865-3868.
[3] a) G. Kresse, D. Joubert, *Phys. Rev. B* **1999**, *59*, 1758-1775; b) P. E. Blöchl, *Phys. Rev. B* **1994**, *50*, 17953-17979.
[4] A. Tkatchenko, M. Scheffler, *Phys. Rev. Lett.* **2009**, *102*, 073005.
[5] J. Tersoff, D. R. Hamann, *Phys. Rev. B* **1985**, *31*, 805-813.
[6] M.-L. Bocquet, H. Lesnard, S. Monturet, N. Lorente, in *Computational Methods in Catalysis and Materials Science* (Eds.: R. A. van Santen, P. Sautet), Wiley-VCH Verlag GmbH & Co. KGaA, **2009**, pp. 199-219.
[7] N. Lorente, R. Robles, v1.0b2 ed., Zenodo, **2019, December 17**.
[8] K. Momma, F. Izumi, *J. Appl. Crystallogr.* **2011**, *44*, 1272-1276.
[9] L. Venkataraman, J. E. Klare, C. Nuckolls, M. S. Hybertsen, M. L. Steigerwald, *Nature* **2006**, *442*, 904-907.
